# Oral administration of Ginsenoside Rg1 prevents cardiac toxicity induced by doxorubicin in mice through anti-apoptosis

**DOI:** 10.18632/oncotarget.19698

**Published:** 2017-07-31

**Authors:** Chen Zhu, Yi Wang, Hua Liu, Haiman Mu, Yue Lu, Jiayi Zhang, Jianhua Huang

**Affiliations:** ^1^ First Affiliated Hospital of Jinzhou Medical University, Jinzhou, China; ^2^ Graduated School of Jinzhou Medical University, Jinzhou, China; ^3^ Third Affiliated Hospital of Jinzhou Medical University, Jinzhou, China

**Keywords:** Rg1, cardiac toxicity, doxorubicin, apoptosis

## Abstract

Although Ginsenoside Rg1 has been reported to have protective cardiac effects, its effects on cardiac toxicity induced by doxorubicin needs to be studied. The present study investigated the effects of oral administration of Rg1 on the heart in mice treated with doxorubicin and found improved fractional shortening and ejection fraction of the heart and decreased cardiac apoptosis in mice treated with doxorubicin. The underlying mechanisms include increased phosphorylation of Akt and Erk by Rg1, increased ratio of Bcl-2 and Bax, and decreased release of cytochrome c from mitochondria, thereby protecting the heart from doxorubicin-induced apoptosis. This phenotype suggested that the oral administration of Rg1 may be a potential method preventing the cardiac toxicity caused by doxorubicin in clinical practice.

## INTRODUCTION

Doxorubicin, an anthracycline antibiotic, is a regularly used chemotherapeutic agent for the treatment of solid tumors, lymphoma, and leukemia. However, it exerts severe side-effects on the heart and results in cardiomyopathy and/or congestive heart failure in cancer patients. This limits its application in cancer patients in clinical practice [1, 2]. The oxidative damage of cardiomyocytes and the consequent death of these cells by apoptosis are the primary characteristics of cellular damage induced by doxorubicin [2, 3]. Various agents such as antioxidants [3], metal chelators [4], angiotensin-converting enzyme [5], and beta-blockers [6] have been applied to prevent the cardiac toxicity caused by doxorubicin with some degree of success. Recently, medicinal plants have been reported to successfully prevent the cardiac toxicity induced by doxorubicin [7–9]. Thus, identifying natural compounds derived from other plants that could prevent the cardiac toxicity of doxorubicin is critical for enhancing the chemotherapeutic efficiency of doxorubicin.

*Panax ginseng*, a well-known traditional Chinese medicine, is widely used due to its promising healing and restorative properties as well as the tonic effect. The consumption of the medication is safe and non-toxic in animals and humans. The major active components of *Panax ginseng* are Ginsenosides, including Rg1, Rg3, Rh1, Re, and Rd. There have been reports that Ginsenoside Rg3 and s-Rh2 can prevent doxorubicin-induced cardiac toxicity [10, 11]. However, the content of both Rg3 and s-Rh2 is extremely lower in *Panax ginseng*, which restricted their broad application in clinical practice. Ginsenoside Rg1, abundant in *Panax ginseng*, has a rigid steroidal skeleton with four transfused rings and two sugar moieties [12]. Rg1 is one of the most active ingredients in *Panax ginseng* and possesses a broad spectrum of activities. Rg1 exhibits estrogen-like activity and may represent a novel class of potent phytoestrogens [13]. Rg1 can activate the glucocorticoid receptor [14]. Rg1 has been demonstrated to increase angiogenesis [15] and enhance the angiogenic potency of endothelial progenitor cells [16]. Recently, several studies showed that Rg1 protected the heart from ischemic and reperfusion injury, decreased the infarct size of the heart and prevented heart remodeling in various animal models [17–19]. However, whether Rg1 prevents the cardiac toxicity caused by doxorubicin needs to be studied.

In this study, we investigated whether Rg1 prevents doxorubicin-mediated cardiac toxicity induced by oral administration. Rg1 was found to prevent cardiac toxicity induced by doxorubicin through anti-apoptosis, which indicated a potential clinical usage of Rg1 in protecting the heart from doxorubicin-induced toxicity.

## RESULTS

### Oral administration of Rg1 preserved the cardiac function in mice treated with doxorubicin in both early and late phase

**Figure 1:**
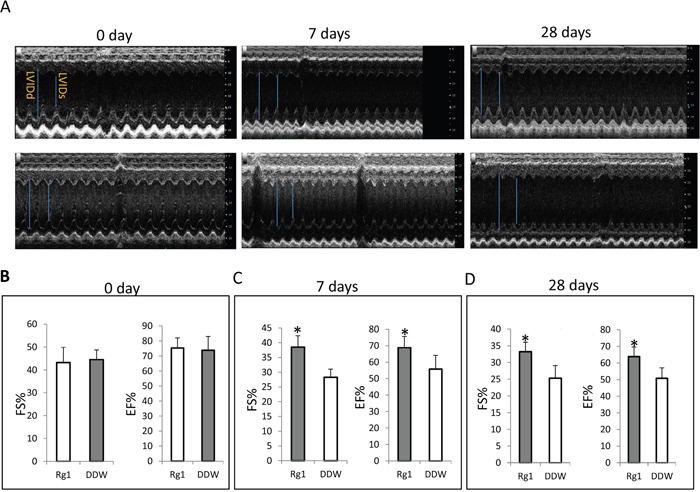
Oral administration of Rg1 preserved the cardiac function in mice treated with doxorubicin on day 7 and 28 **(A)** Echocardiography of the heart. Upper panel, oral administration of Rg1; lower panel, oral administration of DDW. LVIDd: left ventricle internal diameter in diastole; LVIDs: left ventricle internal diameter in systole. **(B)** On day 0, there was no difference of FS and EF between Rg1 and DDW group. **(C)** Oral administration of Rg1 significantly improve FS as compared to the DDW control on day 7, n = 5, ^*^p < 0.05; and oral administration of Rg1 significantly improves EF as compared to the DDW control on day 7, n = 5, ^*^p < 0.01. **(D)** Oral administration of Rg1 significantly improved FS as compared to the DDW control on day 28, n = 5, ^*^p < 0.05; and oral administration of Rg1 significantly improves EF as compared to the DDW control on day 28, n = 5, ^*^p < 0.01.

We selected day 7 and day 28 after doxorubicin treatment as the time points for early and late phase injury by doxorubicin to the heart respectively. Echocardiography was used to detect the effects of oral administration of Rg1 on the cardiac function in mice treated with doxorubicin. On day 7 and 28 after doxorubicin treatment, the oral administration of Rg1 significantly improved the fractional shortening (FS) and ejection fraction (EF) as compared to the DDW control (Figure 1). This indicated that the oral administration of Rg1 preserved the cardiac function in mice treated with doxorubicin in both early and late phase.

### Oral administration of Rg1 decreased serum biochemical markers of cardiac injury in mice treated with doxorubicin

**Figure 2:**
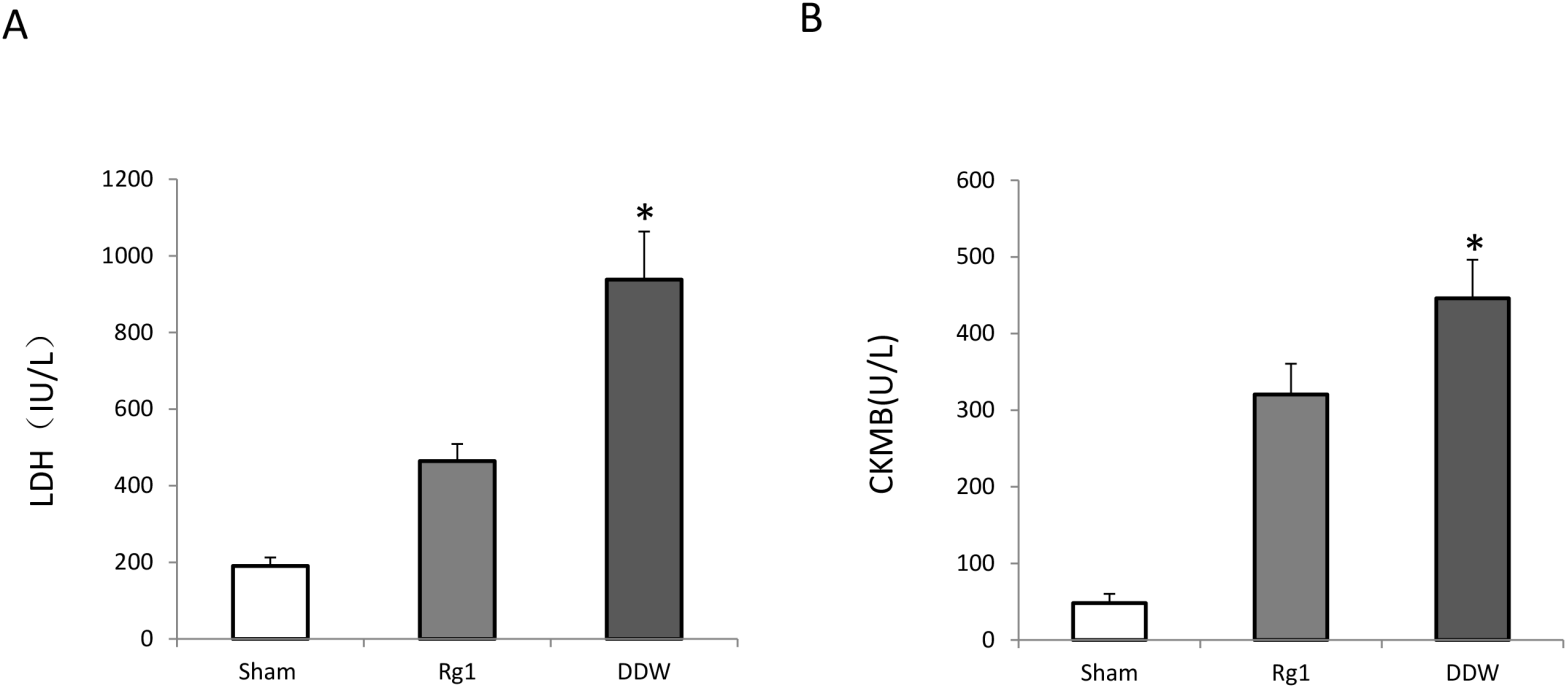
Oral administration of Rg1 decreased CKMB and LH released from the heart in mice treated with doxorubicin on day 7 **(A)** Oral administration of Rg1 significantly decreased LH released from the heart in mice treated with doxorubicin, n = 5, ^*^p < 0.01. **(B)** Oral administration of Rg1 significantly decreased CKMB releasing from the heart in mice treated with doxorubicin, n = 5, ^*^p < 0.01.

To further evaluate the effects of Oral administration of Rg1 on cardiac injury in mice treated with doxorubicin in early phase, the lactate dehydrogenase (LDH) and Creatine kinase MB (CKMB) were detected on day 7 after doxorubicin treatment. The results showed that Oral administration of Rg1 significantly decreased the LDH and CKMB releasing as compared to the DDW control (Figure 2). This indicated that the oral administration of Rg1 decreased cardiac injury in mice treated with doxorubicin in early phase.

### Oral administration of Rg1 inhibited the inflammation and fibrosis of heart in mice treated with doxorubicin

**Figure 3:**
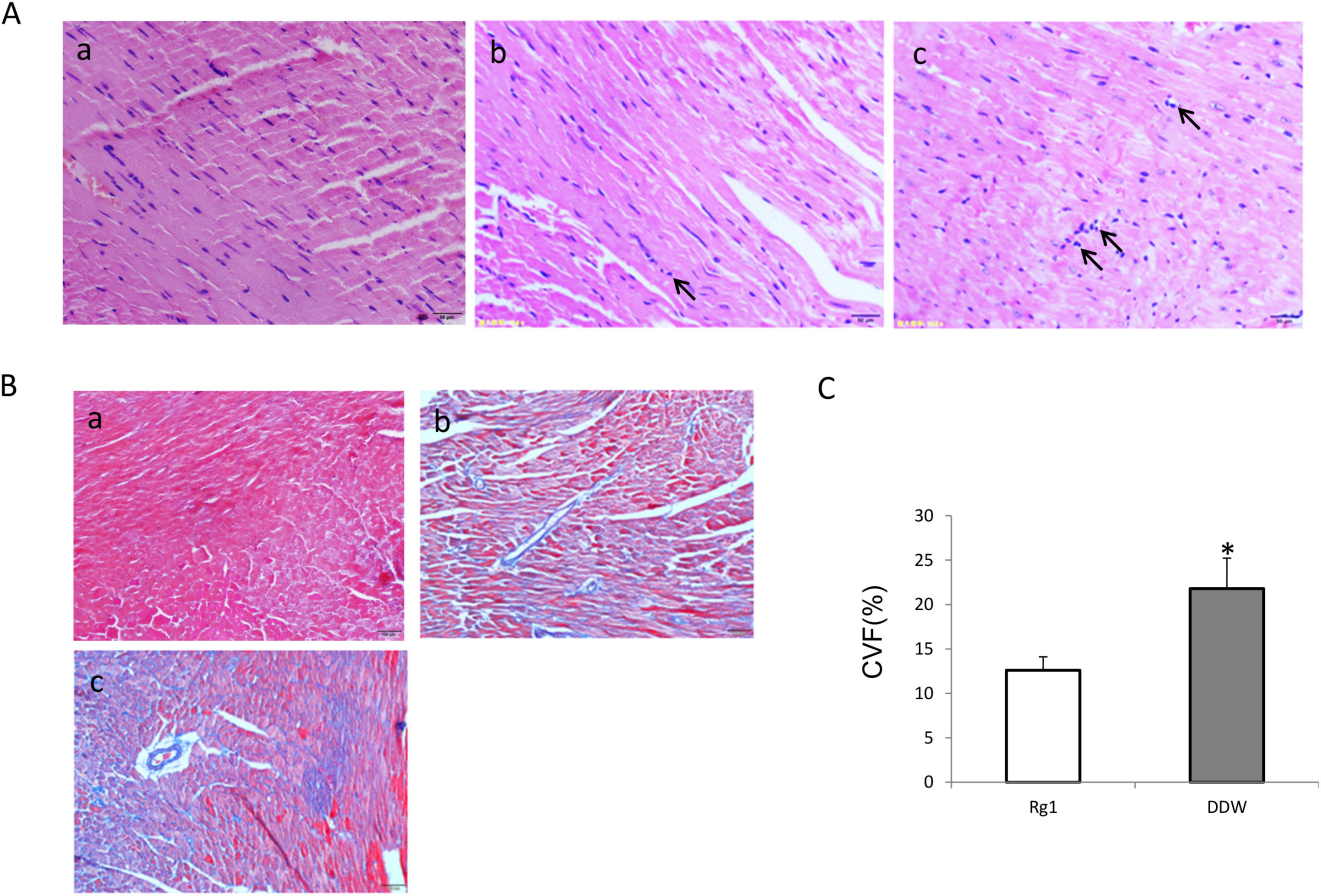
Oral administration of Rg1 inhibited the inflammation and fibrosis of the heart in mice treated with doxorubicin on day 28 **(A)** HE staining of the heart. a: sham; b: heart treated with Rg1; c: heart treated with DDW. Bar = 50 μm. Oral administration of Rg1 distinctly decreased the infiltration of inflammatory cells. Arrows indicated inflammatory cells in the interstitial of the cardiac cells. **(B)** Masson's staining of the heart. a: sham; b: heart treated with Rg1; c: heart treated with DDW. Bar = 100 μm. **(C)** Oral administration Rg1 significantly decreased the fibrosis of heart as compared to the DDW control, n = 5, ^*^p < 0.01.

To further elucidate the effects of oral administration of Rg1 on cardiac injury in mice treated with doxorubicin in late phase, mice were sacrificed on day 28 after doxorubicin treatment, and the hearts were harvested and paraffin embedded. Hematoxylin-eosin (HE) and Masson's staining demonstrated that the oral administration of Rg1 significantly inhibited the infiltration of inflammation of cells into the heart (Figure 3A) and the fibrosis of the heart (Figure 3B and 3C) in mice treated with doxorubicin in late phase.

### Oral administration of Rg1 decreased the apoptosis of cardiac cells in mice treated with doxorubicin

**Figure 4:**
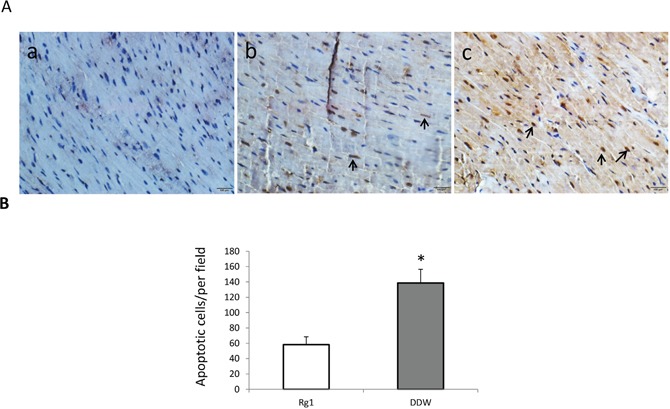
Oral administration of Rg1 decreased the apoptosis in the heart of mice treated with doxorubicin on day 7 **(A)** TUNEL staining of the heart. a: sham; b: heart treated with Rg1; c: heart treated with DDW. Bar = 100 μm. Arrows indicated apoptotic nuclei which were stained brown. **(B)** Oral administration of Rg1 significantly decreased apoptosis of the heart in mice treated with doxorubicin as compared to the DDW control, n = 5, ^*^p < 0.01.

**Figure 5:**
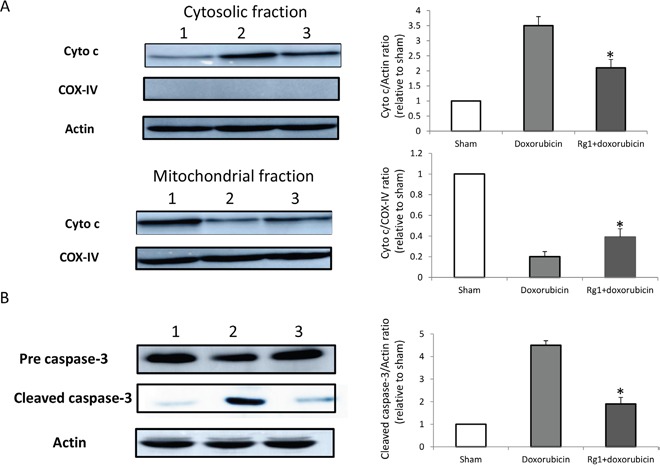
Oral administration of Rg1 inhibited Cyto c release from mitochondria and the cleavage of caspase-3 in the heart of mice treated with doxorubicin on day 7 **(A)** Lane 1: sham; lane 2: DDW control; lane 3: Rg1. Oral administration of Rg1 significantly inhibited Cyto c release from mitochondria as compared to the DDW control, n = 5, ^*^p < 0.01. **(B)** Lane 1: sham; lane 2: DDW control; lane 3: Rg1. Oral administration of Rg1 significantly inhibited cleavage of caspase-3 as compared to the DDW control, n = 5, ^*^p < 0.01.

Inducing cardiac apoptosis is one of the side-effects of doxorubicin on the heart. In order to detect the effects of oral administration of Rg1 on the apoptosis of cardiac cells in mice treated with doxorubicin, we performed TUNEL staining of the heart on day 7 after doxorubicin treatment. The results showed that Rg1 significantly decreased the cardiac apoptosis in mice treated with doxorubicin as compared to the DDW control (Figure 4A and 4B). Furthermore, we found that the oral administration of Rg1 inhibited the release of cytochrome c (Cyto c) from mitochondria (Figure 5A) and decreased the expression of cleaved caspase-3 in the heart of mice treated with doxorubicin, as compared to the DDW control (Figure 5B). These data indicated that anti-apoptosis of the heart was one of the effects, wherein Rg1 preserved the cardiac function in mice treated with doxorubicin.

### Oral administration of Rg1 increased the phosphorylation of Akt and Erk in the heart of mice treated with doxorubicin

**Figure 6:**
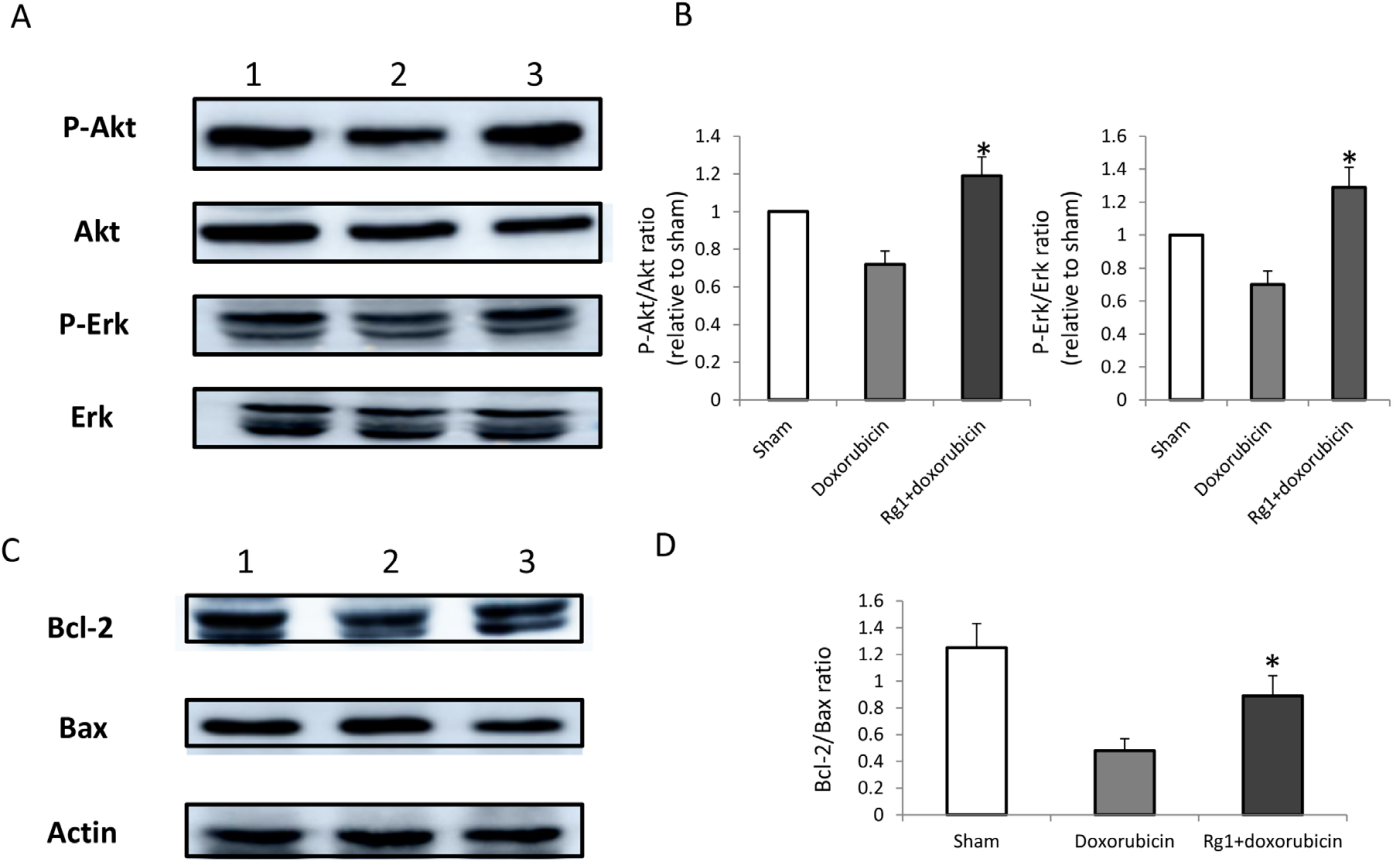
Oral administration of Rg1 increased the phosphorylation of Akt and Erk and the ratio of Bcl-2 and Bax in the heart of mice treated with doxorubicin on day 7 **(A)** Lane 1: sham; lane 2: DDW control; lane 3: Rg1. **(B)** Oral administration of Rg1 significantly increased the phosphorylation of Akt and Erk as compared to the DDW control, n = 5, ^*^p < 0.05. **(C)** Lane 1: sham; lane 2: DDW control; lane 3: Rg1. **(D)** Oral administration of Rg1 significantly increased the ratio of Bcl-2 and Bax in the heart of mice treated with doxorubicin as compared to the DDW control, n = 5, ^*^p < 0.01.

Akt and Erk pathways play a major role in apoptosis. Western blot was performed to detect Akt and Erk in the hearts of mice treated with doxorubicin on day 7 after doxorubicin treatment. We found that the oral administration of Rg1 significantly increased the phosphorylation of Akt and Erk in the hearts of mice treated with doxorubicin (Figure 6A and 6B), which indicated that Akt and Erk plays a crucial role in the anti-apoptosis of cardiac cells by Rg1.

### Oral administration of Rg1 increased the ratio of Bcl-2 and Bax in the heart of mice treated with doxorubicin

Bcl-2 family plays a crucial role in apoptosis. Western blot was performed to detected Bcl-2 and Bax expression in the heart of mice treated with doxorubicin on day 7 after doxorubicin treatment. We found that the oral administration of Rg1 significantly increased the ratio of Bcl-2 and Bax in the hearts of mice treated with doxorubicin as compared to the DDW control (Figure 6C and 6D). This indicated that Rg1 exerts its anti-apoptotic effect by influencing the expression of specific members of the Bcl-2 family.

## DISCUSSION

In the present study, we found that the oral administration of Rg1 decreased the cardiac cell apoptosis caused by doxorubicin and improved the cardiac function. To the best of our knowledge, this is the first time that Rg1 is reported to prevent cardiac toxicity caused by doxorubicin in mice.

Although the mechanisms of cardiac toxicity caused by doxorubicin are not fully elucidated, inducing cardiac apoptosis is one of the side-effects of doxorubicin on the heart. In addition to the nucleus, doxorubicin has also been observed to accumulate in the mitochondria [20]. Mitochondria play a vital role in the process of apoptosis by releasing Cyto c from mitochondria to the cytoplasm to activate the caspases. Doxorubicin-induced oxidative stress and abnormally high levels of calcium in the cell stimulate the release of Cyto c and initiate the apoptotic pathways through caspase activation [21]. Doxorubicin may also promote apoptosis by affecting topoisomerase 2β in the mitochondria, a specific target of doxorubicin [22]. Recent studies suggested that doxorubicin-induced cardiotoxicity may not be solely due to the reactive oxygen species (ROS) produced during the redox cycling reactions of doxorubicin. In the presence of topoisomerase 2β, doxorubicin activates the DNA response genes and consequently the apoptotic pathways. These features further trigger the marked alterations in the transcriptome, which selectively affect the oxidative phosphorylation and mitochondrial biogenesis in cardiomyocytes, leading to mitochondrial oxidative stress and metabolic failure [23, 24]. Furthermore, the derivatives of doxorubicin promote Cyto c release by accumulating in the inner mitochondrial membrane to disrupt the electron transport chain [25]. Herein, we found that doxorubicin significantly increased the Cyto c release from the mitochondria in the heart of mice.

Thus, the strategy against the mitochondrial pathway of apoptosis is reasonable in preventing the cardiac toxicity caused by doxorubicin. Reportedly, the cardiac toxicity caused by doxorubicin is decreased in various animal models by the antiapoptotic methods [26–28]. Several studies showed that Rg1 protects the heart from ischemic reperfusion injury or heart infarction by the effect of anti-apoptosis [17–19]. This phenomenon indicated that Rg1 might also reduce the doxorubicin-induced side-effects to the heart by anti-apoptosis. In the present study, we found that the oral administration of Rg1 significantly improved the cardiac function caused by doxorubicin in mice, and cardiac apoptosis was significantly decreased. Furthermore, we observed that Rg1 increased the phosphorylation of Akt and Erk. This is in accordance with the studies demonstrating the Rg1-mediated a rise in angiogenesis by increasing the phosphorylation of Akt in endothelial cells [14], and in turn, protecting the endothelial cell from apoptosis by increasing the phosphorylation of Erk [29]. PI3K/Akt and mitogen-activated protein kinase (MAPK) are the two key intracellular signaling transduction pathways that participate in various biological activities such as apoptosis and autophagy [30, 31]. Previous studies have illustrated that the activation of Akt and downstream signaling molecules, such as mammalian target of rapamycin (mTOR) and Bad and/or inhibition of p38 MAPK, are capable of inhibiting the doxorubicin-induced cardiac injury [32–34]. Thus, the activation of Akt and Erk pathways is among the mechanisms through which Rg1 prevents the cardiac toxicity effectuated by doxorubicin.

The balance of pro- and antiapoptotic proteins of the Bcl-2 family is known to regulate cell survival and apoptosis. In the mitochondrial apoptosis pathway, both proapoptotic and antiapoptotic proteins of the Bcl-2 family play a major role in regulating the mitochondrial function [35]. Bcl-2 is one of the principal proteins of the Bcl-2 family that protects the cells from apoptosis. It combines with Bax, a proapoptotic protein, to prevent its oligomerization. The oligomeric form of Bax promotes the loss of mitochondrial membrane integrity and causes the release of Cyto c [36]. The stoichiometry of pro- *vs*. antiapoptotic Bcl-2 family members in the cell determines the cell viability [37]. Moreover, doxorubicin decreases the ratio of Bcl-2 and Bax, which is a pivotal mechanism underlying apoptosis [38]. Furthermore, we also found that the oral administration of Rg1 increased the ratio of Bcl-2 and Bax, which resulted in the inhibition of Cyto c mitochondrial release and a subsequent decrease in cardiac apoptosis caused by doxorubicin. This suggested that disrupting the balance of pro- and antiapoptotic proteins of the Bcl-2 family is another critical mechanism through which Rg1 prevents the cardiac toxicity induced by doxorubicin.

Although we proved that the oral administration of Rg1 protected the heart from doxorubicin-induced apoptosis, the apoptosis between cardiac myocytes and endothelial cells was not clearly differentiated. Since doxorubicin is also toxic to the endothelial cells, protecting them is an important aspect of preventing the heart from damage by doxorubicin. Several studies have shown that Rg1 exerts an anti-apoptotic effect on endothelial cells, and thus, the protective effects of Rg1 against the toxicity to endothelial cells caused by doxorubicin in the heart should be investigated further.

In summary, we found that the oral administration of Rg1 preserves the cardiac function, and decreases cardiac apoptosis caused by doxorubicin. These phenotypes are governed by Rg1 activating the Akt and Erk pathway by increasing their phosphorylation and increased the ratio of Bcl-2 and Bax, thereby preventing the cardiac apoptosis caused by the toxicity of doxorubicin. This suggested that Rg1 might serve as a putative tool for preventing the cardiac toxicity caused by doxorubicin in clinical practice.

## MATERIALS AND METHODS

### Reagents

Rg1 was purchased from the National Institute for Food and Drug Control (Beijing, China). Doxorubicin was procured from Aladdin Industrial Company (Shanghai, China, purity 98%). Anti-p-Akt (Ser473), anti-Akt, anti-p-Erk (Thr202/Tyr204), anti-Erk, anti-Bcl-2, anti-Bax, anti-cleaved caspase-3, anti-caspase-3, anti-Cyto c, anti-Cyto c oxidase IV (COX-IV) and anti-β-actin were obtained from Cell Signaling Technology (Shanghai, China).

### Animals

All animal experiments were approved by the Experimental Animal Ethics Committee of Jinzhou Medical University and conformed to the Guide for the Care and Use of Laboratory Animals published by the USA National Institutes of Health (Publication, 8^th^ Edition, 2011). Healthy male C57BL/6 mice (weight 20–25g) were purchased from Beijing Vital River Laboratory Animal Technology Co., Ltd., Beijing, China. The mice were allowed free access to standard chow and water at room temperature 22–24 °C and 12 h light/dark cycle. The animals were acclimatized for a minimum of 1 week before the experiments.

### Experimental setting

Thirty mice were randomly divided into 3 groups: sham (n = 10), doxorubicin (n = 10), and doxorubicin + Rg1 treatment group (n = 10). In the sham group, double distilled water (DDW) was orally administered daily 1 week before the mice were intraperitoneally injected with DDW containing no doxorubicin one time, and oral administration of DDW was continued until sacrificing mice. In the doxorubicin group, DDW was orally administered daily 1 week before the mice were intraperitoneally injected with doxorubicin in DDW (15 mg/kg) one time, and oral administration of DDW was continued until sacrificing mice. In the doxorubicin + Rg1 group, Rg1 in DDW (80 mg/kg/day) was orally administered daily 1 week before the mice were intraperitoneally injected with doxorubicin in DDW (15 mg/kg) one time, and oral administration of Rg1 was continued until sacrificing mice. Because 7 days mimic acute doxorubicin exposure [39] and 28 days mimic chronic doxorubicin exposure [40], we selected 7 days and 28 days as the time points for the early and late injury to the heart by doxorubicin respectively. Echocardiography was performed to evaluate cardiac function immediately before doxorubicin treatment and on day 7 and 28 after doxorubicin treatment. For the detection of effects of Rg1 on the early injury to the heart by doxorubicin, 5 mice in each group were sacrificed on day 7; the blood samples were collected for the detection of serum biochemical markers of cardiac injury; the hearts were harvested for TUNEL staining and western blot analysis of apoptosis-related protein. For the detection of effects of Rg1 on the late injury to the heart by doxorubicin, the remaining mice (n = 5 in each group) were sacrificed on day 28, the hearts were harvested for histological analysis of changes in morphological pathology.

### Echocardiography

Echocardiography was performed to measure the cardiac function by Prospect High-Resolution Imaging System (S-Sharp Corporation, Taiwan, China) immediately before doxorubicin treatment, on day 7 and 28 after doxorubicin treatment in all mice. After anesthetized with inhalation of 1.0% isoflurane, the mice were placed in a supine position on the platform with heating to maintain the body temperature. The heart was imaged in the 2-D mode in the parasternal short-axis view with a depth setting of 2 cm. The M-mode cursor (40 MHz) was positioned perpendicular to interventricular septum and posterior wall of left ventricle at the level of papillary muscles from the 2-D mode. The sweep speed was 100mm/s for the M-mode. Left ventricular end diastolic dimension and left ventricular systolic dimension were measured. EF and FS were calculated and selected as the indexes of the cardiac function. The value of EF and FS averaged from at least 3 separate cardiac cycles.

### LDH and CKMB measurements

Five mice in each group were sacrificed on day 7 after doxorubicin treatment, and the blood samples were withdrawn immediately through the right atrium. After standing at room temperature for 30 min, the samples were centrifuged (3000 rpm, 10 min), and serum samples were collected. LDH and CKMB were evaluated by a full-automatic biochemical detection machine (COBAS C 311, Roche Diagnostics GmbH, Germany) using specific LDH and CKMB detection kits (BIOBASE, Jinan, Shangdong, China). The procedure was performed following manufacture's manual.

### HE and Masson's trichrome staining

Five mice in each group were sacrificed on day 28 after doxorubicin treatment, and cardiac samples were harvested, embedded in paraffin, and sliced into 4-μm-thick sections. Subsequently, the sections were stained with HE or Masson's trichrome for histological and collagen analysis, respectively. Five images were randomly selected and photographed under microscope in each section of individual heart. The interstitial fibrotic areas were assessed by image analysis software (Image-pro plus 6.0; Media Cybernetics LP, Washington, USA). The collagen volume fraction (CVF) was calculated as the ratio of collagen area to total area.

### TUNEL assay

The cardiac samples were harvested from mice sacrificed on day 7 after doxorubicin treatment (n = 5 in each group). Heart was cut into 2 parts from middle of the ventricle. One part of the heart was embedded in paraffin, and sliced into 4-μm-thick sections. *In situ* Cell Death Detection Kit (Roche, Indianapolis, USA) was used to detect the apoptosis in the heart of mice according to the manufacture's protocol. After washing three times with PBS, the treated samples were fixed in 4% paraformaldehyde for 1 h and permeabilized in 0.1% Triton X-100 sodium citrate buffer for 2 min. Then, the sections were randomly selected for terminal dUTP nick end-labeling (TUNEL), and the apoptotic cells were revealed using the diaminobenzidine (DAB) kit. Nuclei were counterstained with hematoxylin. The total number of TUNEL-positive nuclei was enumerated in 5 randomly chosen fields of view per tissue section in a blinded manner, and the results were expressed as the number of TUNEL-positive nuclei/field.

### Western blot

The remaining part of the heart from mice sacrificed on day 7 after doxorubicin treatment were collected (n = 5 in each group). For the detection of protein in the whole cell lysate, the remaining heart tissue was futher cut into 2 pieces, one piece of heart tissue was placed in 0.4 mL cold lysis buffer containing 20 mmol/L HEPES (pH 7.5), 150 mmol/L NaCl, 1 mmol/L EDTA, 0.5% Triton X-100, and protease inhibitors (Roche) and homogenized with a mixing homogenizer (Kinematica AG). To estimate the Cyto C released from the mitochondria, the another piece of heart tissue was immersed in 0.4 mL lysis buffer containing 250 mmol/L sucrose, 20 mmol/L HEPES, 10 mmol/L KCl, 1 mmol/L MgCl2, 1 mmol/L EDTA, 1 mmol/L EGTA, 1 mmol/L dithiothreitol, and 1 mmol/L phenylmethylsulfonyl fluoride, pH 7.5 and incubated for 5 min on ice. The heart was homogenized with a mixing homogenizer, and the suspension was centrifuged at 750 ×*g* for 10 min at 4 °C to sediment the nuclear fraction. The supernatant was collected and centrifuged at 12,000 ×*g* for 10 min at 4 °C to sediment the mitochondrial fraction. The resultant supernatant was further centrifuged at 14,000 ×*g* for 10 min at 4 °C and filtered through a 0.22 μm ultrafilter (Millipore) to generate a purified cytosolic fraction. An equivalent of 40 μg total protein extracts was separated by SDS-PAGE, transferred to nitrocellulose membranes, and blocked for 1 h at room temperature. For the detection of protein expression, the membranes were probed overnight at 4 °C with the primary antibodies (dilution 1:1000), followed by anti-mouse or anti-rabbit secondary antibodies (dilution 1:2000) conjugated to horseradish peroxidase (Zymed Inc., San Francisco, CA, USA) for 1 h at room temperature. The enhanced chemiluminescence ECL Plus system (Amersham Biosciences, Little Chalfont, UK) was used to evaluate the immunoreactivity. β-actin (1:1000) was used as an internal control. COX- IV (1:1000) was used as the mitochondria marker. The intensity of the bands was measured with a scanning densitometer (Bio-Rad) coupled with Bio-Rad analysis software.

### Statistical analysis

All the values were presented as mean ± SD. One-way ANOVA followed by Bonferroni/Dunn test was used for group comparisons. A *p*-value < 0.05 was considered statistically significant.

## Abbreviations

LDH: lactate dehydrogenase
CKMB: creatine kinase MB
FS: fractional shortening
EF: ejection fraction
DDW: double distilled water
HE: hematoxylin-eosin
Cyto c: cytochrome c
ROS: reactive oxygen species
CVF: collagen volume fraction

## References

[1] Weidner C, Rousseau M, Plauth A, Wowro S, Fischer C, Abdel-Aziz H, Sauer S (2015). Melissa officinalis extract induces apoptosis and inhibits proliferation in colon cancer cells through formation of reactive oxygen species. Phytomedicine.

[2] Thorn CF, Oshiro C, Marsh S, Hernandez-Boussard T, McLeod H, Klein TE, Altman RB (2011). Doxorubicin pathways: Pharmacodynamics and adverse effects. Pharmacogenet Genomics.

[3] Wouters KA, Kremer L, Miller TL, Herman EH, Lipshultz SE (2005). Protecting against anthracycline-induced myocardial damage: a review of the most promising strategies. Brit J haematol.

[4] Yagmurca M, Fadillioglu E, Erdogan H, Ucar M, Sogut S, Irmak MK (2003). Erdosteine prevents doxorubicin-induced cardiotoxicity in rats. Pharmacol Res.

[5] Hiona A, Lee AS, Nagendran J, Xie X, Connolly AJ, Robbins RC, Wu JC (2011). Pretreatment with angiotensin-converting enzyme inhibitor improves doxorubicin-induced cardiomyopathy via preservation of mitochondrial function. J Thorac Cardiovasc Surg.

[6] Tashakori Beheshti A, Mostafavi Toroghi H, Hosseini G, Zarifian A, Homaei Shandiz F, Fazlinezhad A (2016). Carvedilol administration can prevent doxorubicin-induced cardiotoxicity: a double-blind randomized trial. Cardiology.

[7] Yilmaz S, Atessahin A, Sahna E, Karahan I, Ozer S (2006). Protective effect of lycopene on adriamycin-induced cardiotoxicity and nephrotoxicity. Toxicology.

[8] Hamza A, Amin A, Daoud S (2008). The protective effect of a purified extract of withania somnifera against doxorubicin-induced cardiac toxicity in rats. Cell Biol Toxicol.

[9] Li W, Xu B, Xu J, Wu XL (2009). Procyanidins produce significant attenuation of doxorubicin-induced cardiotoxicity via suppression of oxidative stress. Basic Clin Pharmacol Toxicol.

[10] Wang X, Chen L, Wang T, Jiang X, Zhang H, Li P, Lv B, Gao X (2015). Ginsenoside Rg3 antagonizes adriamycin-induced cardiotoxicity by improving endothelial dysfunction from oxidative stress via upregulating the Nrf2-ARE pathway through the activation of akt. Phytomedicine.

[11] Wang H, Yu P, Gou H, Zhang J, Zhu M, Wang ZH, Tian JW, Jiang YT, Fu FH (2012). Cardioprotective effects of 20(S)-ginsenoside Rh2 against doxorubicin-induced cardiotoxicity in vitro and in vivo. Evid Based Complement Alternat Med.

[12] Ling C, Li Y, Zhu X, Zhang C, Li M (2005). Ginsenosides may reverse the dexamethasone-induced down-regulation of glucocorticoid receptor. Gen Comp Endocrinol.

[13] Chan RY, Chen WF, Dong A, Guo D, Wong MS (2002). Estrogen-like activity of ginsenoside Rg1 derived from Panax notoginseng. J Clin Endocrinol Metab.

[14] Leung KW, Pon YL, Wong RN, Wong AS (2006). Ginsenoside-Rg1 induces vascular endothelial growth factor expression through the glucocorticoid receptor-related phosphatidylinositol 3-kinase/Akt and beta-catenin/T-cell factor-dependent pathway in human endothelial cells. J Biol Chem.

[15] Yang N, Chen P, Tao Z, Zhou N, Gong X, Xu Z, Zhang M, Zhang D, Chen B, Tao Z (2012). Beneficial effects of ginsenoside-Rg1 on ischemia-induced angiogenesis in diabetic mice. Acta Biochim Biophys Sin (Shanghai).

[16] Shi A, Gu N, Liu X, Wang X, Peng Y (2011). Ginsenoside rg1 enhances endothelial progenitor cell angiogenic potency and prevents senescence in vitro. J Int Med Res.

[17] Dong G, Chen T, Ren X, Zhang Z, Huang W, Liu L, Luo P, Zhou H (2016). Rg1 prevents myocardial hypoxia/reoxygenation injury by regulating mitochondrial dynamics imbalance via modulation of glutamate dehydrogenase and mitofusin 2. Mitochondrion.

[18] Deng Y, Yang M, Xu F, Zhang Q, Zhao Q, Yu H, Li D, Zhang G, Lu A, Cho K, Teng F, Wu P, Wang L (2015). Combined salvianolic acid B and ginsenoside Rg1 exerts cardioprotection against ischemia/reperfusion injury in rats. PLoS One.

[19] Lim KH, Lim DJ, Kim JH (2013). Ginsenoside-Re ameliorates ischemia and reperfusion injury in the heart: a hemodynamics approach. J Ginseng Res.

[20] Nicolay K, Fok JJ, Voorhout W, Post JA, de Kruijff B (1986). Cytofluorescence detection of adriamycin-mitochondria interactions in isolated, perfused rat heart. Biochim Biophys Acta.

[21] Octavia Y, Tocchetti CG, Gabrielson KL, Janssens S, Crijns HJ, Moens AL (2012). Doxorubicin-induced cardiomyopathy: from molecular mechanisms to therapeutic strategies. J Mol Cell Cardiol.

[22] Low RL, Orton S, Friedman DB (2003). A truncated form of DNA topoisomerase IIbeta associates with the mtDNA genome in mammalian mitochondria. Eur J Biochem.

[23] Hao E, Mukhopadhyay P, Cao Z, Erdelyi K, Holovac E, Liaudet L, Lee WS, Hasko G, Mechoulam R, Pacher P (2015). Cannabidiol protects against doxorubicin-induced cardiomyopathy by modulating mitochondrial function and biogenesis. Mol Med.

[24] Zhang S, Liu X, Bawa-Khalfe T, Lu LS, Lyu YL, Liu LF, Yeh ET (2012). Identification of the molecular basis of doxorubicin-induced cardiotoxicity. Nat Med.

[25] Thorn CF, Oshiro C, Marsh S, Hernandez-Boussard T, McLeod H, Klein TE, Altman RB (2011). Doxorubicin pathways: pharmacodynamics and adverse effects. Pharmacogenet Genomics.

[26] Hamza AA, Ahmed MM, Elwey HM, Amin A (2016). Melissa officinalis protects against doxorubicin-induced cardiotoxicity in rats and potentiates Its anticancer activity on MCF-7 Cells. PLoS One.

[27] Wang L, Zhang TP, Zhang Y, Bi HL, Guan XM, Wang HX, Wang X, Du J, Xia YL, Li HH (2016). Protection against doxorubicin-induced myocardial dysfunction in mice by cardiac-specific expression of carboxyl terminus of hsp70-interacting protein. Sci Rep.

[28] Junkun L, Erfu C, Tony H, Xin L, Sudeep KC, Mingliang Z, Yanqin W, XiangQian Q (2016). Curcumin downregulates phosphate carrier and protects against doxorubicin induced cardiomyocyte apoptosis. Biomed Res Int.

[29] Yan J, Liu Q, Dou Y, Hsieh Y, Liu Y, Tao R, Zhu D, Lou Y (2013). Activating glucocorticoid receptor-ERK signaling pathway contributes to ginsenoside Rg1 protection against β-amyloid peptide-induced human endothelial cells apoptosis. J Ethnopharmacol.

[30] Ceci M, Ross J, Condorelli G Molecular determinants of the physiological adaptation to stress in the cardiomyocyte: a focus on AKT. J Mol Cell Cardiol.

[31] Muslin AJ (2008). MAPK signalling in cardiovascular health and disease: molecular mechanisms and therapeutic targets. Clin Sci (Lond).

[32] Singla DK (2015). Akt-mTOR pathway inhibits apoptosis and fibrosis in doxorubicin-induced cardiotoxicity following embryonic stem cell transplantation. Cell Transplant.

[33] Fan GC, Zhou X, Wang X, Song G, Qian J, Nicolaou P, Chen G, Ren X, Kranias EG (2008). Heat shock protein 20 interacting with phosphorylated Akt reduces doxorubicin-triggered oxidative stress and cardiotoxicity. Circ Res.

[34] Wang X, Wang XL, Chen HL, Wu D, Chen JX, Wang XX, Li RL, He JH, Mo L, Cen X, Wei YQ, Jiang W (2014). Ghrelin inhibits doxorubicin cardiotoxicity by inhibiting excessive autophagy through AMPK and p38-MAPK. Biochem Pharmacol.

[35] Gustafsson AB, Gottlieb RA (2007). Bcl-2 family members and apoptosis, taken to heart. Am J Physiol Cell Physiol.

[36] Mikhailov V, Mikhailova M, Pulkrabek DJ, Dong Z, Venkatachalam MA, Saikumar P (2001). Bcl-2 prevents Bax oligomerization in the mitochondrial outer membrane. J Biol Chem.

[37] Wong WW, Puthalakath H (2008). Bcl-2 family proteins: the sentinels of the mitochondrial apoptosis pathway. IUBMB Life.

[38] Anghel N, Cotoraci C, Ivan A, Suciu M, Herman H, Balta C, Nicolescu L, Olariu T, Galajda Z, Ardelean A, Hermenean A (2015). Chrysin attenuates cardiomyocyte apoptosis and loss of intermediate filaments in a mouse model of mitoxantrone cardiotoxicity. Histol Histopathol.

[39] Montgomery MD, Chan T, Swigart PM, Myagmar BE, Dash R, Simpson PC (2017). An alpha-1A adrenergic receptor agonist prevents acute doxorubicin cardiomyopathy in male mice. PLoS One.

[40] Räsänen M, Degerman J, Nissinen TA, Miinalainen I, Kerkelä R, Siltanen A, Backman JT, Mervaala E, Hulmi JJ, Kivelä R, Alitalo K (2016). VEGF-B gene therapy inhibits doxorubicin-induced cardiotoxicity by endothelial protection. Proc Natl Acad Sci U S A.

